# ﻿A new species and some new distribution records of the genus *Apatetica* Westwood from China (Coleoptera, Staphylinidae, Apateticinae)

**DOI:** 10.3897/zookeys.1212.130072

**Published:** 2024-09-13

**Authors:** Jin-Kang Chang, Harald Schillhammer, Liang Tang

**Affiliations:** 1 College of Life Sciences, Shanghai Normal University, 100 Guilin Road, 1st Educational Building 323 Room, Shanghai, 200234, China Shanghai Normal University Shanghai China; 2 Natural History Museum Vienna, International Research Institute for Entomology, Burgring 7, A - 1010 Wien, Austria Natural History Museum Vienna, International Research Institute for Entomology Wien Austria

**Keywords:** Apateticinae, Guangdong, Guangxi, identification key, Oriental region, Sichuan, Xizang, Yunnan

## Abstract

Five species of *Apatetica* Westwood, 1848 from China are recorded and illustrated: *Apateticaangusticollis***sp. nov.** from Sichuan, *A.confusa* Assing, 2018 from Yunnan, *A.intermedia* Cameron, 1930 from Sichuan, *A.laevicollis* Fauvel, 1904 from Guangdong and Guangxi, and *A.sikkimi* Fauvel, 1895 from Xizang. The last four are new to China. The new species is described, and a photograph of a living specimen is presented. *Apateticaintermedia* and *A.sikkimi* are redescribed and diagnosed. A key to Chinese species of the genus is provided.

## ﻿Introduction

The genus *Apatetica* Westwood, 1848 is known only from East and Southeast Asia. It is one of the two genera composing the small subfamily Apateticinae Fauvel, 1895 (Coleoptera, Staphylinidae) . At present, four *Apatetica* species have been recorded from China: *A.gibba* Assing, 2018 from Gansu, *A.aspera* Assing, 2018 from Sichuan, *A.glabra* Assing, 2018 from Yunnan, and *A.curtipennis* Assing, 2018 from Yunnan. In this study, four new country records and a new species from China are presented. *Apateticaintermedia* Cameron, 1930 and *A.sikkimi* Fauvel, 1895 are redescribed, and photographs of their complete habitus and aedeagi are available for the first time. A comprehensive revision of the entire subfamily with more detailed data is in preparation.

## ﻿Material and methods

The specimens examined in this paper were collected by sifting mixed leaf litter and killed with ethyl acetate. To examine the genitalia, the last two abdominal segments were detached from the body after softening it in hot water. Aedeagi were mounted in Euparal (Chroma Gesellschaft Schmidt, Koengen, Germany) on plastic slides. Photos of sexual characters were taken with a Canon G9 camera attached to an Olympus SZX 16 stereoscope; habitus photos were taken with a Canon MP-E 65 mm macro lens attached to a Canon EOS7D camera and stacked with Zerene Stacker.

The specimens treated in this study are deposited in College of Life Sciences, Shanghai Normal University, China (**SHNU**), Naturhistorisches Museum Wien, Austria (**NMW**), the Natural History Museum, London, U.K. (**BMNH**), the Institut Royal des Sciences Naturelle, Bruxelles, Belgium (**IRSNB**), and the Naturhistoriska Riksmuseet, Stockholm, Sweden (**NHRS**).

The measurements of proportions are abbreviated as follows:

**BL** body length, measured from the anterior margin of the clypeus to the abdominal apex

**FL** forebody length, measured from the anterior margin of the clypeus to the apex of the elytra

**HW** width of head including eyes

**PW** width of pronotum

**EW** width of elytra

**AL** length of antennae

**PL** length of pronotum

**EL** length of elytra, measured at the widest point.

## ﻿Taxonomy

### ﻿ Apatetica angusticollis

Taxon classificationAnimaliaColeopteraStaphylinidae

Chang, Schillhammer & Tang sp. nov.

2A8AA915-DE2E-5DB5-A0DA-943F4880260F

https://zoobank.org/4F55802F-3274-44BE-8B3C-B0199C2ACA81

Figs 1 , 2 狭领脊葬隐翅虫

#### Material examined.

***Holotype.*** China – **Sichuan Prov.** • ♂; glued on a card with labels as follows: “China: Sichuan, Wenchuan County, Zhenghegou; alt. 1456–1615 m; 15 May 2015; Ming-Xia Guo leg; SNCA1111.” “Holotype / *Apateticaangusticollis* / Chang, Schillhammer & Tang” [red label]; SHNU. ***Paratypes.*** China – **Sichuan Prov.** • 1♀, SNCA1114; same data as for holotype; NMW • 2♀♀, SNCA1112, SNCA1113; Dayi County, Xiling Snow Mt.; 30°38'60"N, 103°10'99"E; alt. 1250 m; 31 Jul 2021; Zhao & Cai leg.; SHNU.

**Figure 1.**
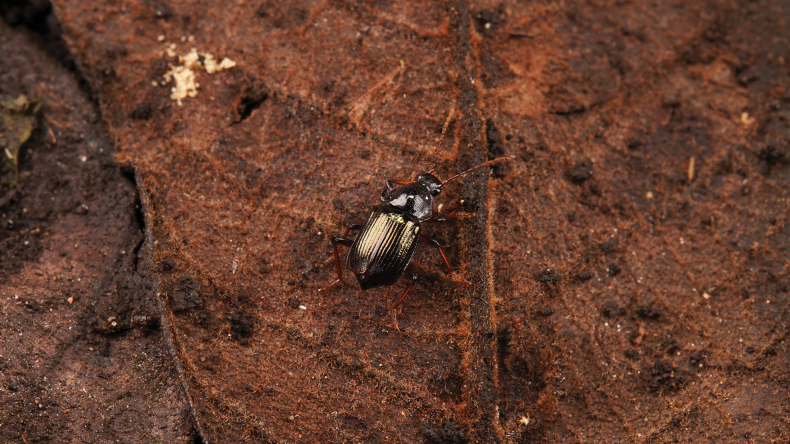
living *Apateticaangusticollis* sp. nov. (SNCA1112) on decaying grass and leaf litter (photo by Mr. Qing-Hao Zhao, taken in Sichuan, Dayi County, Xiling Snow Mt. on 31 Jul. 2021).

#### Description.

Measurements: BL: 7.6–7.9 mm, FL: 7.0–7.3 mm. HW: 1.52–1.76 mm, PW: 2.99–3.38 mm, EW: 3.19–3.68 mm, AL: 4.07–4.12 mm, PL: 1.81–2.16 mm, EL: 3.99–4.26 mm.

**Figure 2.**
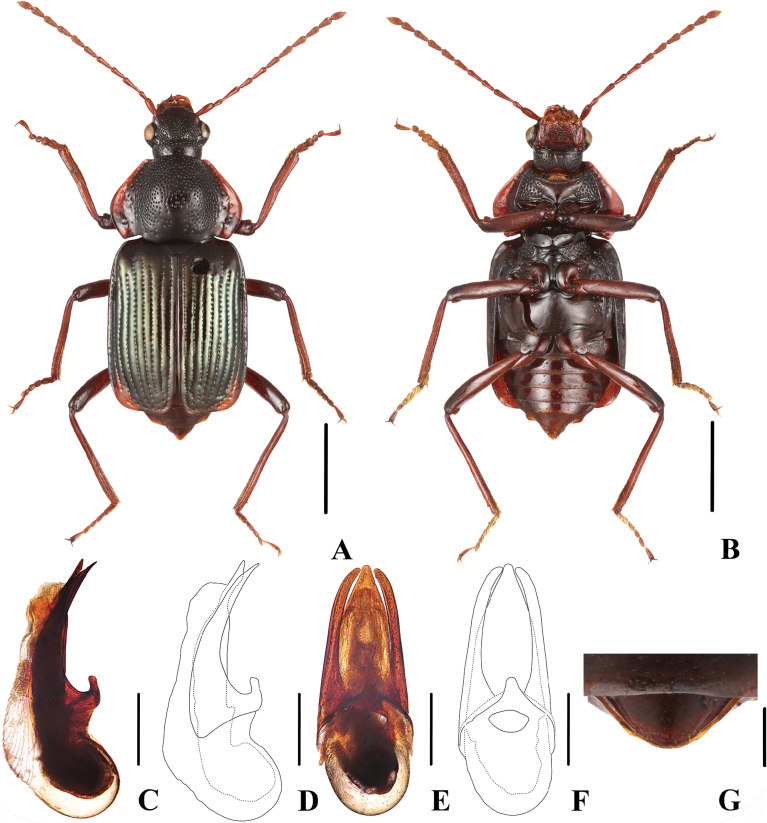
*Apateticaangusticollis* sp. nov. **A–F** SNCA1111 **G** SNCA1112 **A** dorsal habitus **B** ventral habitus **C, D** aedeagus in lateral view **E, F** aedeagus in ventral view **G** female tergite VIII. Scale bars: 2 mm (**A, B**); 0.5 mm (**C–F**); 0.25 mm (**G**).

Body (Fig. 2A, B) blackish, lateral margins of pronotum broadly dark reddish-brown; elytra with metallic-greenish hue; antennae, mouth parts and legs dark reddish-brown, femora becoming darker brown distally.

Head transverse; anterior portion with flat elevation between anterior margin and vertex, sides of elevation glossy; in postero-median portion with a cluster of numerous macropunctures; frons glossy; punctation of portions near inner margin of eye very dense with somewhat elongated punctural grooves. Antennae very long and slender; antennomere 4 approximately 2.5 times as long as broad, 5 three times as long as broad, and 10 approximately twice as long as broad.

Pronotum approximately 1.6 times as broad as long and 2.0 times as broad as head, broadest in posterior third; anterior margin narrower than head; lateral margins broadly explanate, gradually becoming narrower in anterior half, each with several marginal punctures; disc moderately elevated; punctation of disc coarse, dense laterally and sparser in middle; postero-median portions of disc with irregular impunctate patches.

Elytra approximately 2.1–2.2 times as long as pronotum; each elytron with nine moderately coarsely punctate striae; intervals flat with micropunctation; lateral apical angles of elytra obtuse. Scutellum weakly transverse. Legs very long and slender.

Abdomen with microsculpture on tergites VI–VIII.

**Male.** Tergite VIII convex, wedge-shaped; aedeagus (Fig. 2C–F) 1.8 mm long; median lobe robust, gradually tapering apicad, with apex moderately obtuse in ventral view; parameres moderately long and slender, apically tapered, and curved ventrad.

**Female.** Tergite VIII (Fig. 2G) relatively flat, apically rounded.

#### Distribution.

China (Sichuan).

#### Diagnosis.

The new species can be recognized among all known congeners with metallic elytra by the combination of the following characters: antennae and most of the legs reddish brown, pronotum with anterior margin narrower than head, and elytra with obtuse lateral apical angles.

#### Etymology.

The specific epithet is an adjective composed of the Latin adjective angustus (narrow) and the Latin noun collum (the neck). It alludes to the narrow anterior margin of the pronotum.

### ﻿ Apatetica confusa

Taxon classificationAnimaliaColeopteraStaphylinidae

Assing, 2018

A3139B64-4A6E-5414-9AF9-57FFF89F4970

Fig. 3 蛊惑脊葬隐翅虫

Apatetica confusa Assing, 2018: 351.

#### Material examined.

China – **Yunnan Prov.** • 4♂♂, 3♀♀, SNCA1117–SNCA1123; Gongshan Hsien, Dulongjiang Country, Maku Village; 27°40'57"N, 98°18'08"E; alt. 1250 m; 24 Apr 2015; Wen-Xuan Bi leg.; SHNU.

**Figure 3.**
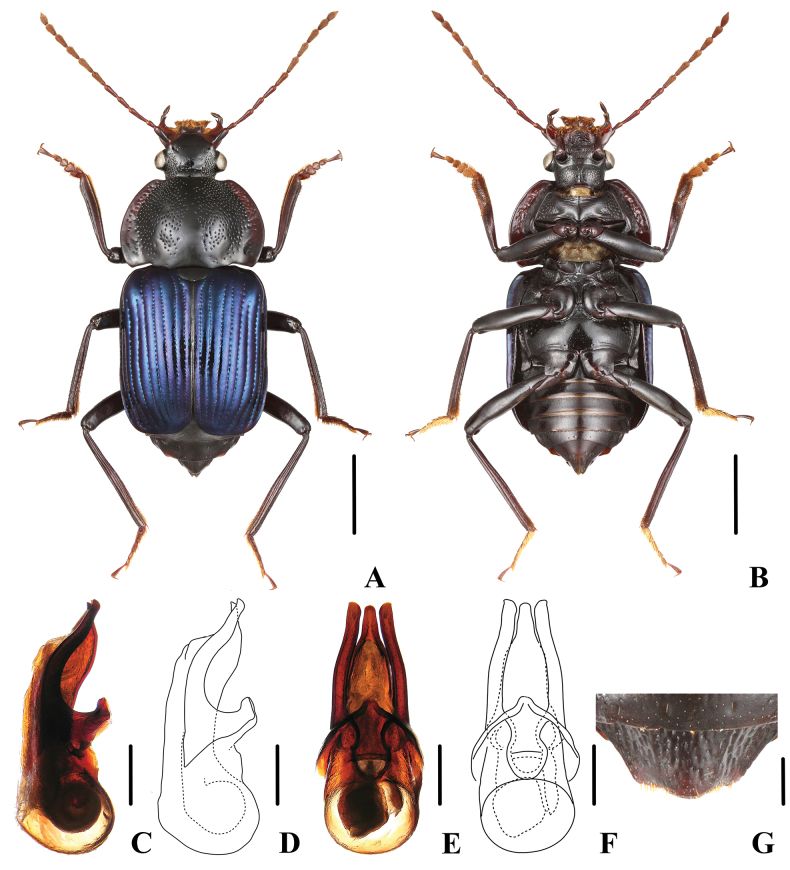
*Apateticaconfusa***A–F** SNCA1117 **G** SNCA1118 **A** dorsal habitus **B** ventral habitus **C, D** aedeagus in lateral view **E, F** aedeagus in ventral view **G** female tergite VIII. Scale bars: 2 mm (**A, B**); 0.5 mm (**C–F**); 0.25 mm (**G**).

#### Measurements.

BL: 8.4–9.1 mm, FL: 7.3–8.1 mm. HW: 1.76–1.89 mm, PW: 3.58–3.77 mm, EW: 3.63–3.82 mm, AL: 4.56–4.90 mm, PL: 2.16–2.38 mm, EL: 4.17–4.36 mm.

#### Distribution.

China (Yunnan) and Myanmar. New to China.

#### Remarks.

The metallic hue of the elytra ranges from usually greenish-coppery to rarely blue or violet (Assing 2018). The specimens we collected in Yunnan all have blue metallic elytra. New record for China.

### ﻿ Apatetica intermedia

Taxon classificationAnimaliaColeopteraStaphylinidae

Cameron, 1930

39030E46-5E6E-5859-9D1F-BC7634C656FE

Fig. 4 中型脊葬隐翅虫

Apatetica intermedia Cameron, 1930: 30; Madge 1980: 317.
Apatetica birmanica Jansson, 1946: 7.

#### Type material examined.

*A.intermedia*: There is one specimen in the BMNH from the type locality with the following label data: “60853 \ Doherty \ Birmah Ruby M^es^ \ Fry coll. 1905 100. \ Apateticaintermedia Cam. i.l.“. It is not specifically labeled as the type, but it may be the type specimen.

*A.birmanica*: ***Holotype*** ♂: “N.E. BURMA, Kambaiti, 2000 m, 4/6.1934 *Malaise* \ Typus \ Apateticabirmanica Jansson \ 6378 E91 \ NHRS-JLKB 000027515” (NHRS); 2 ***paratypes*** (sex not determined): “N.E. Burma, Kambaiti, 7000 ft. 25-27/4 1934, R. MALAISE \ Paratypus \ 6376 [6377 resp.] E91 \ NHRS-JLKB 000027541 [000027516 resp.]” (NHRS).

#### Material examined.

China – **Sichuan Prov.** • 3♂♂, 3♀♀, SNCA1158–SNCA1163; Dayi County, Xiling Snow Mt.; 30°38'60"N, 103°10'99"E; alt. 1250 m; 31 Jul 2021; Zhao & Cai leg.; SHNU.

#### Measurements.

BL: 8.2–8.3 mm, FL: 6.4–6.7 mm. HW: 1.57–1.67 mm, PW: 3.23–3.43 mm, EW: 3.53–3.68 mm, AL: 2.84–2.89 mm, PL: 1.81–1.96 mm, EL: 3.58–3.77 mm.

#### Redescription.

**Figure 4.**
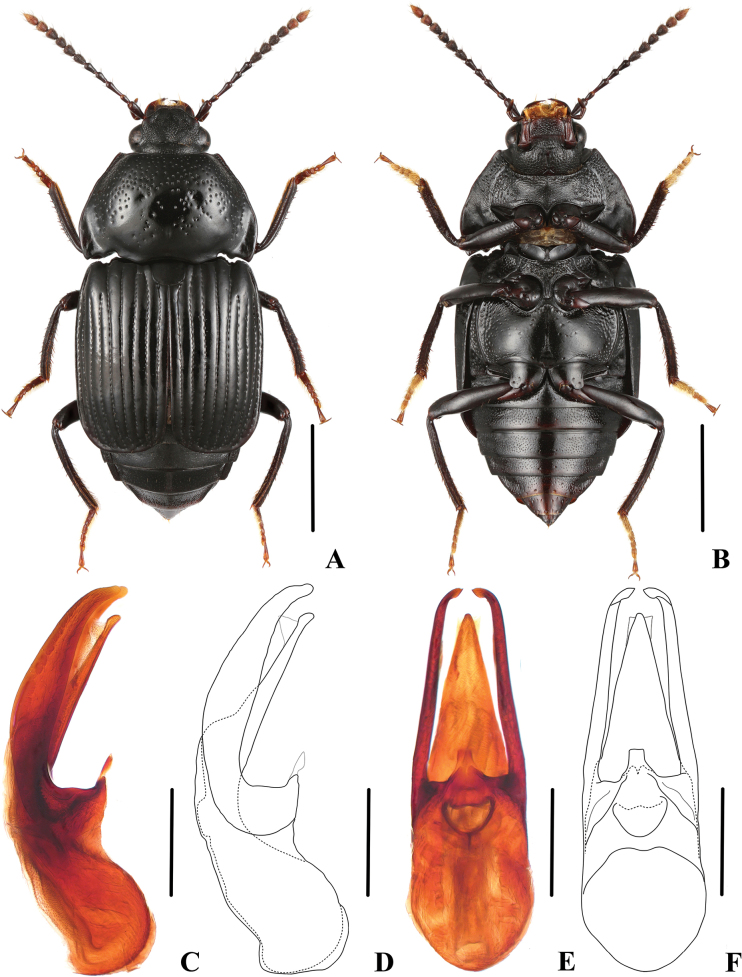
*Apateticaintermedia* (SNCA1158) **A** dorsal habitus **B** ventral habitus **C, D** aedeagus in lateral view **E, F** aedeagus in ventral view. Scale bars: 2 mm (**A, B**); 0.5 mm (**C–F**).

(based on specimens from Sichuan). Body (Fig. 4A, B) entirely black, except antennomeres 10 and 11, and tarsi slightly reddish.

Head transverse; vertex with a pair of small punctate patches separated by a cluster of numerous punctures; punctation of portions near inner margin of eye very dense and elongated; interstices with distinct microsculpture. Antennae moderately long; antennomere 4, 5 approximately 1.8 times as long as broad, 6–10 weakly oblong.

Pronotum approximately 1.8 times as broad as long and 2.1 times as broad as head, broadest in posterior third, strongly tapering anteriad; explanate part of lateral margins narrow in anterior half, gradually becoming broader in posterior half; each side of the posterior margin with a tooth-like extension, exactly fitting into an emargination at base of elytra; anterior angles with weak projection, posterior angles sharply marked and directed posteriad; disc convex in cross-section, with a pair of groove-like dents at middle, punctation coarse and quite dense in anterior third, irregularly distributed in median third and almost absent in basal third.

Elytra short, 1.9–2.0 times as long as pronotum; anterior margin of shoulders with an abrupt excavation opposite the pronotal basal tooth; each elytron with nine finely punctate striae; intervals rather flat and with micropunctation; posterior margin of each elytron convex. Scutellum weakly transverse with transverse microsculpture. Legs short.

Abdomen with microsculpture, punctation dense and moderately coarse.

**Male.** Tergite VIII convex, proximally broad, apically obtusely pointed; aedeagus (Fig. 4C–F) 1.7 mm long; median lobe moderately slender, gradually tapering apicad, and apically moderately acute in ventral view; apical portion of ventral process straight in lateral view; parameres long and weakly slender, extending beyond apex of median lobe and curved ventrad apically.

**Female.** Tergite VIII flat, wedge-shaped, apically obtusely pointed.

#### Distribution.

China (Sichuan) and Myanmar. New to China.

#### Diagnosis.

*Apateticaintermedia* can be distinguished from all known congeners, except *A.sikkimi* Fauvel, 1895, by the anterior margin of shoulders which have an abrupt excavation opposite the pronotal basal tooth. *Apateticaintermedia* differs from *A.sikkimi* by the relatively larger eyes, the weak projection on the anterior angles of pronotum (pronotal anterior angles with no projection in *A.sikkimi*), and relatively sparser pronotal punctation.

### ﻿ Apatetica laevicollis

Taxon classificationAnimaliaColeopteraStaphylinidae

Fauvel, 1904

283B77CB-C2EF-5098-9211-0415C1223EBE

Fig. 5 光胸脊葬隐翅虫

Apatetica laevicollis Fauvel, 1904: 86; Madge 1980: 315; Assing 2018: 354.

#### Material examined.

China – **Guangdong Prov.** • 3♂♂, 4♀♀, SNCA1171–SNCA1177; Shixing County, Chebaling Mt., Xianrendong Village; 24°44'05"N, 114°12'26"E; alt. 508 m; 23–26 Jul 2008; Liang H. B. leg.; SHNU. • 2♀♀, SNCA1178, SNCA1179; Shixing County, Chebaling Mt., Chebaling Village; 24°40'55"N, 114°11'49"E; alt. 590 m; 24 Jul 2008; Liang H. B. leg.; SHNU. – **Guangxi Prov.** • 2♂♂, 4♀♀, SNCA1164–SNCA1169; Xing’an County, Maoershan Mt.; 25°53'11"N, 110°28'13"E; beech forest, mixed leaf litter, humus, sifted; alt. 810 m; 28 Jul 2014; Peng, Song, Yu & Yan leg.; SHNU. • 1♀, SNCA1170; Jinxiu County, Laoshan Forest Farm; 24°07'02"N, 110°11'51"E; beech forest, mixed leaf litter, humus, sifted; alt. 950 m; 26 Jul 2014; Peng, Song, Yu & Yan leg.; SHNU.

**Figure 5.**
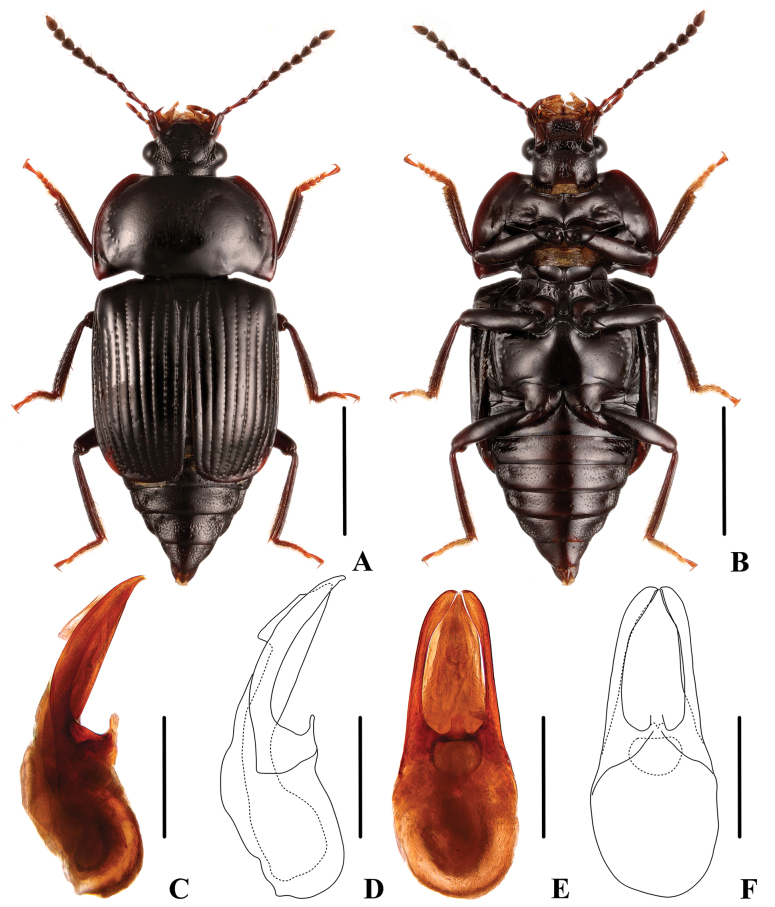
*Apateticalaevicollis* (SNCA1164) **A** dorsal habitus **B** ventral habitus **C, D** aedeagus in lateral view **E, F** aedeagus in ventral view. Scale bars: 2 mm (**A, B**); 0.5 mm (**C–F**).

#### Measurements.

BL: 7.6–8.1 mm, FL: 6.1–6.3 mm. HW: 1.39–1.42 mm, PW: 3.03–3.19 mm, EW: 3.09–3.19 mm, AL: 2.77–2.84 mm, PL: 1.67–1.72 mm, EL: 3.38–3.48 mm.

#### Distribution.

China (Guangdong and Guangxi) and Vietnam. New to China.

#### Remarks.

The previous record from the Chinese province Sichuan (Madge 1980) is based on a misidentification and refers to *A.gibba*. New record for China.

### ﻿ Apatetica sikkimi

Taxon classificationAnimaliaColeopteraStaphylinidae

Fauvel, 1895

BF21BBF7-71AF-537E-99B8-1FF13910EF7F

Fig. 6 锡金脊葬隐翅虫

Apatetica sikkimi Fauvel, 1895: 193; Madge 1980: 317.
Apatetica indica Cameron, 1930: 29.

#### Type material examined.

*A.sikkimi*: ***Holotype*** (sex not determined): “Darjeeling \ sikkimi Fvl. \ I.R.Sc.N.B. 17.479 Apatetica Coll. et det. A. Fauvel“ (IRSNB).

*A.indica*: ***Holotype*** (sex not determined): “British Bootang, Maria Basti, 1899 \ Oberthur Coll. 1902-65 \ Apateticaindica Cam. Type \ Determined from description G.J.A. Apateticasikkimi, Fauv. \ 2FF coll. Oberth. 2 MM from same locality agree with A. viridipennis, Fauv.“ (BMNH).

#### Remark.

The type locality of *A.indica* is in West Bengal, India. Maria Basti is located in Kalimpong environs in Darjeeling.

#### Material examined.

China – **Xizang Prov.** • 1♂, 1♀, SNCA1153, SNCA1155; Hanmi; alt. 2100 m; 12–31 Jul 2013; Wen-Xuan Bi leg.; SHNU. • 1♂, SNCA1154; Motuo County, forest nr. 80K; 29°39'29"N, 95°29'23"E; alt. 2100 m; 11–12 Aug 2022; Peng, Song, Yin & Zhang leg.; SHNU.

#### Measurements.

BL: 7.4–9.7 mm, FL: 6.3–7.6 mm. HW: 1.42–1.62 mm, PW: 3.19–3.58 mm, EW: 3.38–3.72 mm, AL: 2.70–3.38 mm, PL: 1.86–2.06 mm, EL: 3.48–4.12 mm.

#### Redescription.

**Figure 6.**
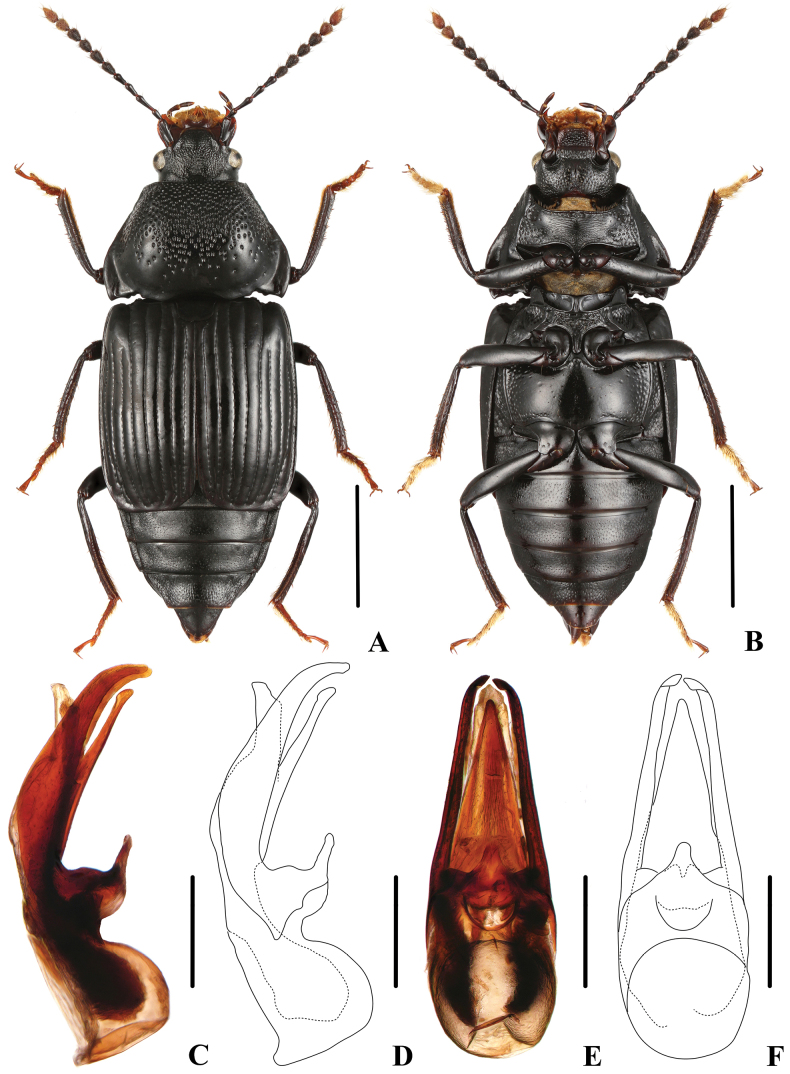
*Apateticasikkimi* (SNCA1153) **A** dorsal habitus **B** ventral habitus **C, D** aedeagus in lateral view **E, F** aedeagus in ventral view. Scale bars: 2 mm (**A, B**); 0.5 mm (**C–F**).

(based on specimens from Xizang). Body (Fig. 6A, B) entirely black, except antennomeres 10, 11 and tarsi slightly reddish.

Head weakly transverse, eyes small; dorsal surface rather flat, with punctation coarse, dense, and partly confluent. Antennae moderately long; antennomere 4 twice as long as broad, 5 nearly twice as long as broad, 6–8 weakly oblong, and 10 approximately as long as broad.

Pronotum approximately 1.7 times as broad as long and 2.2 times as broad as head, broadest in posterior fourth, strongly tapering anteriad; explanate part of lateral margins extremely narrow in anterior half, barely visible, gradually becoming broader in posterior half; each side of the posterior margin with a tooth as in *A.intermedia*; anterior angles with no projection, posterior angles sharply marked and directed posteriad; disc steeply convex in cross-section, with punctation conspicuously coarse and very dense in antero-median portion, slightly less dense in lateral portions and almost absent in basal quarter.

Elytra short, 1.9–2.0 times as long as pronotum; anterior margin of shoulders with an abrupt excavation opposite the pronotal basal tooth; each elytron with nine finely punctate striae; intervals flat and with micropunctation; posterior margins truncate in the middle. Scutellum weakly transverse and with transverse microsculpture. Legs short.

Abdomen with microsculpture, punctation dense and moderately coarse.

**Male.** Tergite VIII convex, proximally broad, apically obtusely pointed; aedeagus (Fig. 6C–F) 1.7 mm long; median lobe moderately slender, gradually tapering apicad, with apex moderately acute in ventral view; apical portion of ventral process straight in lateral view; parameres long and moderately slender, extending beyond apex of median lobe and strongly curved ventrad apically.

**Female.** Tergite VIII flat, wedge-shaped, apically obtusely pointed.

#### Distribution.

Bhutan, China (Xizang) and India. New to China.

#### Diagnosis.

Except for *A.intermedia* (the difference is mentioned above), the species is similar to *A.curtipennis*, but it can be recognized from latter by the smaller eyes, pronotal anterior angles without a projection (*A.curtipennis*: anterior angles with prominent projection), the distribution of pronotal large punctation (*A.curtipennis*: punctation conspicuously coarse, very dense in antero-median portion, slightly less dense in lateral portions, and somewhat irregularly distributed in posterior portion) and anterior margin of shoulders with an abrupt excavation opposite the pronotal basal tooth (*A.curtipennis*: shoulders with no excavation).

### ﻿Key to species of *Apatetica* from China

**Table:** 

1	Antennae long, more than half the body length; elytra with metallic hue	**2**
–	Antennae short, much less than half the body length; elytra without metallic hue	**3**
2	Anterior margin of pronotum narrower than head	***A.angusticollis* sp. nov. China (Sichuan)**
–	Anterior margin of pronotum wider than head	***A.confusa* China (Yunnan), Myanmar**
3	Pronotal disc with punctation fine and regularly distributed	**4**
–	Pronotal disc with punctation coarse and irregularly distributed	**6**
4	Pronotum completely black, more convex	***A.laevicollis* China (Guangdong, Guangxi), Vietnam**
–	Lateral margins of the pronotum dark reddish-brown, pronotum less convex	**5**
5	Body smaller, length 6.6–6.8 mm; frons, vertex and pronotum with microsculpture	***A.gibba* China (Gansu)**
–	Body larger, length 7.4–8.2 mm; frons, vertex and pronotum without microsculpture	***A.glabra* China (Yunnan)**
6	Pronotal disc with irregular gibbosities, pronotal punctation extremely coarse	***A.aspera* China (Sichuan)**
–	Pronotal disc without gibbosities	**7**
7	Base of elytra with distinct emargination next to shoulder	**8**
–	Base of elytra without distinct emargination	***A.curtipennis* China (Yunnan)**
8	Pronotum with moderately dense punctation in anterior portion and with prominent anterior angles	***A.intermedia* China (Sichuan), Myanmar**
–	Pronotum with extremely dense punctation in anterior portion without prominent anterior angles	***A.sikkimi* Bhutan, China (Xizang), India**

## Supplementary Material

XML Treatment for Apatetica angusticollis

XML Treatment for Apatetica confusa

XML Treatment for Apatetica intermedia

XML Treatment for Apatetica laevicollis

XML Treatment for Apatetica sikkimi

## ﻿Additional information

### Author ORCIDs

Jin-Kang Chang https://orcid.org/0009-0000-2946-6507

Harald Schillhammer https://orcid.org/0000-0001-6888-7953

Liang Tang https://orcid.org/0000-0002-6731-4544

### Data availability

All of the data that support the findings of this study are available in the main text.
